# Quality of life and oral health in elderly

**DOI:** 10.4317/jced.53317

**Published:** 2016-12-01

**Authors:** Víctor-Manuel Paredes-Rodríguez, Gema Torrijos-Gómez, José González-Serrano, Rosa-María López-Pintor-Muñoz, Miguel-Ángel López-Bermejo, Gonzalo Hernández-Vallejo

**Affiliations:** 1 DDS, PhD. Collaborator Professor. Faculty of Odontology. Complutense University of Madrid. Department of Stomatology III; 2 DDS. Collaborator Professor. Faculty of Odontology. Complutense University of Madrid. Department of Stomatology IV; 3 DDS. Master Student. Faculty of Odontology. Complutense University of Madrid. Department of Stomatology III; 4 DDS, PhD. Associate Professor. Faculty of Odontology. Complutense University of Madrid. Department of Stomatology III; 5 MD, DDS, PhD. Head Professor. Faculty of Odontology. Complutense University of Madrid. Department of Stomatology IV; 6 MD, DDS, PhD. Head Professor. Faculty of Odontology. Complutense University of Madrid. Department of Stomatology III

## Abstract

**Background:**

We want to assess quality of life in elderly patients in relation to the number of remaining teeth, the number of ingested drugs and xerostomía and to determine the correlation between an increased intake of drugs and a greater feeling of dry mouth and to know the most commonly used measures to control xerostomia.

**Material and Methods:**

30 subjects aged between 65 and 95 years (14 males, 16 females) completed the OHIP questionnaire to determine quality of life. For oral status, the number of remaining teeth according to WHO criteria and xerostomia using the xerostomia index (XI) were studied. In cases of dry mouth sensation, the measures to alleviate it were asked.

**Results:**

The average quality of life according to the OHIP rate is 19.23 (Dt = 10.58), being 56 the worst quality of life. The Pearson correlation coefficient indicates that quality of life is not related to the number of remaining teeth (r = -0.046; *p* = 0.810) nor the number of ingested drugs (r = 0.226; *p* = 0.23) but a greater sensation of dry mouth is related to a poorer quality of life (r = 0.678; *p* = 0.230). There is no association between the number of ingested drugs and the xerostomia index (r = 0.144; *p* = 0.447). The most frequently measures used against dry mouth were drinking water (21 subjects) and sugarless candies (15 subjects).

**Conclusions:**

Quality of life is not related to the number of remaining teeth nor the number of ingested drugs. However, a higher level of xerostomia was significantly associated with a poorer quality of life. There is no association between the number of drugs ingested and xerostomia index. Sugarless candies and drinking water are the more frequently used measures to alleviate dry mouth.

** Key words:**Quality of life, oral health, elderly.

## Introduction

People over 60 constitute the fastest growing age group worldwide, increasing from 7.9% in 1950 to 14.3% in 2000. In fact, this age group is expected to triple in the next four decades, from 739 million in 2009 to 2000 million in 2050 (1,2). In Europe, the percentage of people older than 65 has increased over the last 50 years, representing 14.7% of the European population. Currently, in the EU there are 13.9 million people older or equal to 65 and 6 million people over 80 years old. It is estimated that the population aged 65 or more and 80 or older will increase to 29.2 and 39% respectively by 2050 (3).

Since health is the most important requirement for elderly people to enjoy their last years of life; both government agencies and health care providers have started to implement policies and programs to promote health to improve the quality of life of this sector of the population (2).

Aging is a complex biological phenomenon that results from an interaction between genetic and environmental factors. This process may directly or indirectly increase the risk of developing diseases (4). Aging may develop a large number of pathological and/or physiological changes that could influence dental treatments. As people get older, more local and/or systemic diseases are diagnosed, which require the use of medication. Furthermore, the use of drugs (about 75% of adults over 60 use at least one medication) may endanger oral health (1,5).

Epidemiological data related to oral health of the elderly are scarce or nonexistent (5). Oral problems encountered in these patients are tooth loss, periodontal disease, tooth decay and oral cancer. The prevalence of edentulism varies between 6% and 78% depending on the society studied (6).

People with dentures have less bite force. They need more time to chew and the masticatory efficiency is reduced between 16-50% as well as the speed of mandibular movements (6). This can make the elderly consume fewer fruits, vegetables and meat, preferring soft foods that are high in saturated fat and cholesterol (6-8).

Xerostomia is common in the geriatric patient with a prevalence ranging between 12-28%, which increase to 40-60% in institutionalized persons. Dry mouth causes serious problems in oral tissues as well as in oral function: increased incidence of caries, gingivitis, difficulty in chewing, swallowing, speech and taste, burning sensation, etc. (6,9). Being polymedicated is the most common cause of xerostomia, since more than 400 drugs can cause it (9,10).

WHO defines quality of life as ‘individuals’ perceptions of their position in life in the context of the culture and value systems in which they live and in relation to their goals, expectations, standards and concerns’. Thus, several factors could influence quality of life, including oral health (11). Some diseases and health problems may affect the general welfare of individuals, which would justify the development of instruments for measuring quality of life (12). Oral Health Related Quality of Life (OHRQoL) is defined as an individual´s assessment of how functional factors, psychological factors, social factors and experience of pain/discomfort in relation to orofacial concerns affect their well-being (13).

The Oral Health Impact Profile (OHIP) is the most used questionnaire in order to assess OHRQoL. It consists of 49 items (OHIP-49) that evaluate seven dimensions of OHRQoL: functional lchological disability, social disability and handicap (12,14). However, its implementation may take a long time, so Slade has developed a shortened version of OHIP with a total of 14 items (OHIP-14), which measures the same dimensions, being the most widely research test used at the present time (12).

Most studies conducted up to date evaluating quality of life related to oral disease, pain and / or discomfort are related to negative experiences, suggesting that patients with any of these three things have a poorer quality of life. Moreover, most of the elderly patients take drugs, which is often associated with xerostomia. The objectives of this study are: (i) to evaluate the quality of life in elderly patients in relation to the number of remaining teeth, the number of ingested drugs and xerostomia; (ii) to determine the correlation between an increased intake of drugs with a greater feeling of dry mouth and (iii) to know the most commonly measures used to control xerostomia.

## Material and Methods

-Sample: Geriatric subjects institutionalized at the residence “La Casa más Grande” (Huecas, Toledo. Spain) were evaluated to perform this cross-sectional study. All patients included in the study signed an informed consent. The inclusion criteria was (i) to be over 65 years old, (ii) not to be bedridden and (iii) to be in full mental conditions to answer the questionnaires by themselves. People who did not consent and those with cognitive impairment were excluded from the study.

From an initial population of 40 individuals, 8 were excluded for not meeting inclusion criteria. Of the remaining 32 only 30 agreed to participate in the study. The variables age, sex and number of drugs daily taken were collected.

-Questionnaires

**Figure 1:**
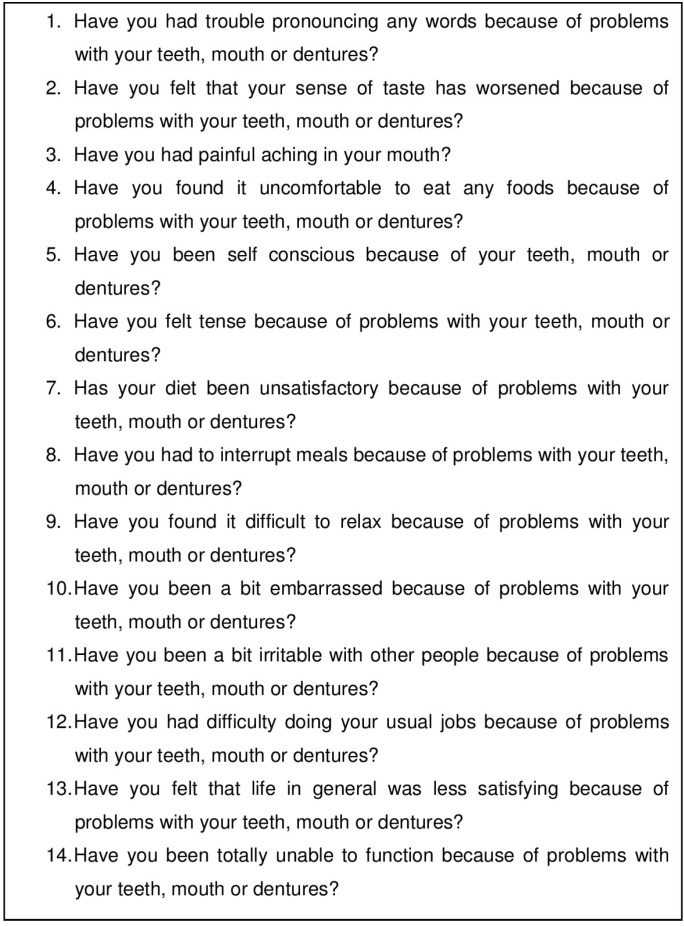
OHIP-14 questionnaire.

•Quality of life: To measure quality of life the OHIP-14 questionnaire was used. It consists of 14 items that describe health aspects related to quality of life in a context of oral health (Fig. 1).

•Oral status: The number of remaining teeth and xerostomia was studied. Teeth were recorded according to WHO criteria. Root fragments were considered as missing teeth. Dry mouth was clasified using the translated version of the Xerostomia index (XI), which is a rating scale of 11 items that results in a single continuous score representing the severity of xerostomia.

•Dental habits: Dental habits were registered based on: (i) the last visit to the dentist (3 months, 6 months or more than 6 months), (ii) autonomous brushing or (iii) attended brushing. When the questionnaire showed the patient had dry mouth, we asked whether any measures were used to palliate it: nothing, sugarless candies, sugar candies, dry mouth pills, special toothpaste, chewing-gum, drink (water, coffee, juice).

-Statistical Analysis: A descriptive analysis of all variables was performed. The Pearson correlation coefficient was used to analyze the possible linear association between quality of life with age, number of drugs taken, number of remaining teeth, xerostomia, as well as between the number of drugs taken and xerostomia. The relationship between quality of life and dichotomous variables sex, visiting the dentist, autonomous brushing and attended brushing was performed using a test for two independent samples. Previously, the Shapiro-Wilk test was applied to study the normality of both groups in quality of life variable. In non-normal samples nonparametric Mann-Whitney test was performed. In all other cases, t Student test was applied. Finally, the relationship between quality of life and the type of prosthesis was studied using the nonparametric Kruskal-Wallis test. Values ≤0.05 were considered as significant. The SPSS software was used for data analysis (version 21 for Windows).

## Results

30 subjects with an average age of 80.50 ± 8.93 (46.7% male and 53.3% female) were studied.

**Figure 2:**
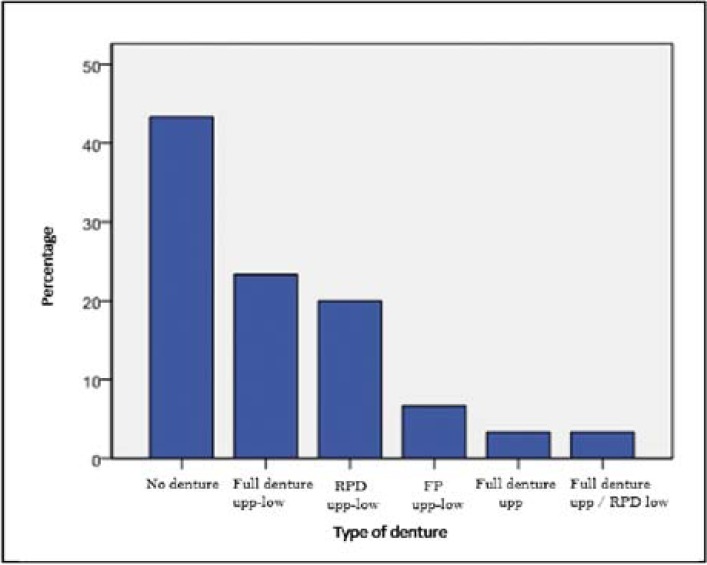
Bar chart: type of denture.

-Dental health and quality of life: The descriptive statistical variables of dental health are presented in  Table 1 and  Table 2. It is observed that most of the subjects did not use dentures (Fig. 2). Most patients went to the dentist every more than six months. Almost every elder was able to brush their teeth by themselves. The average quality of life according to the OHIP-14 index was 19.23 ± 10.58. After applying the Pearson correlation coefficient, no significant relationship between quality of life and age or number of drugs taken was found ( Table 3). No significant relationship between sex and level of quality of life was found. We also found no association between quality of life and frequency of dental visits. Finally, it was noticed that the type of prosthesis is independent of the quality of life.

**Table 1:**
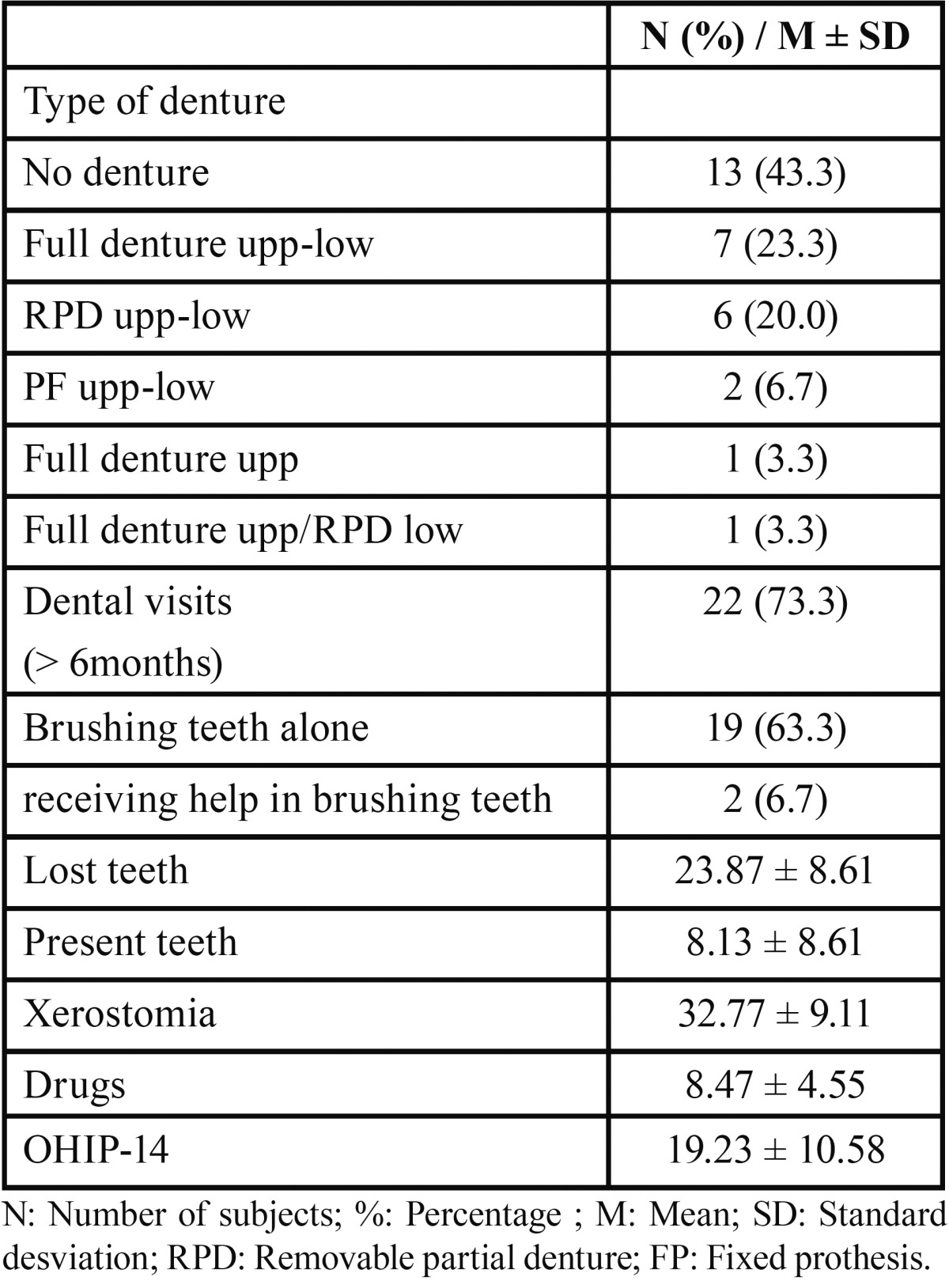
Descriptive statistics of variables dental health.

**Table 2:**
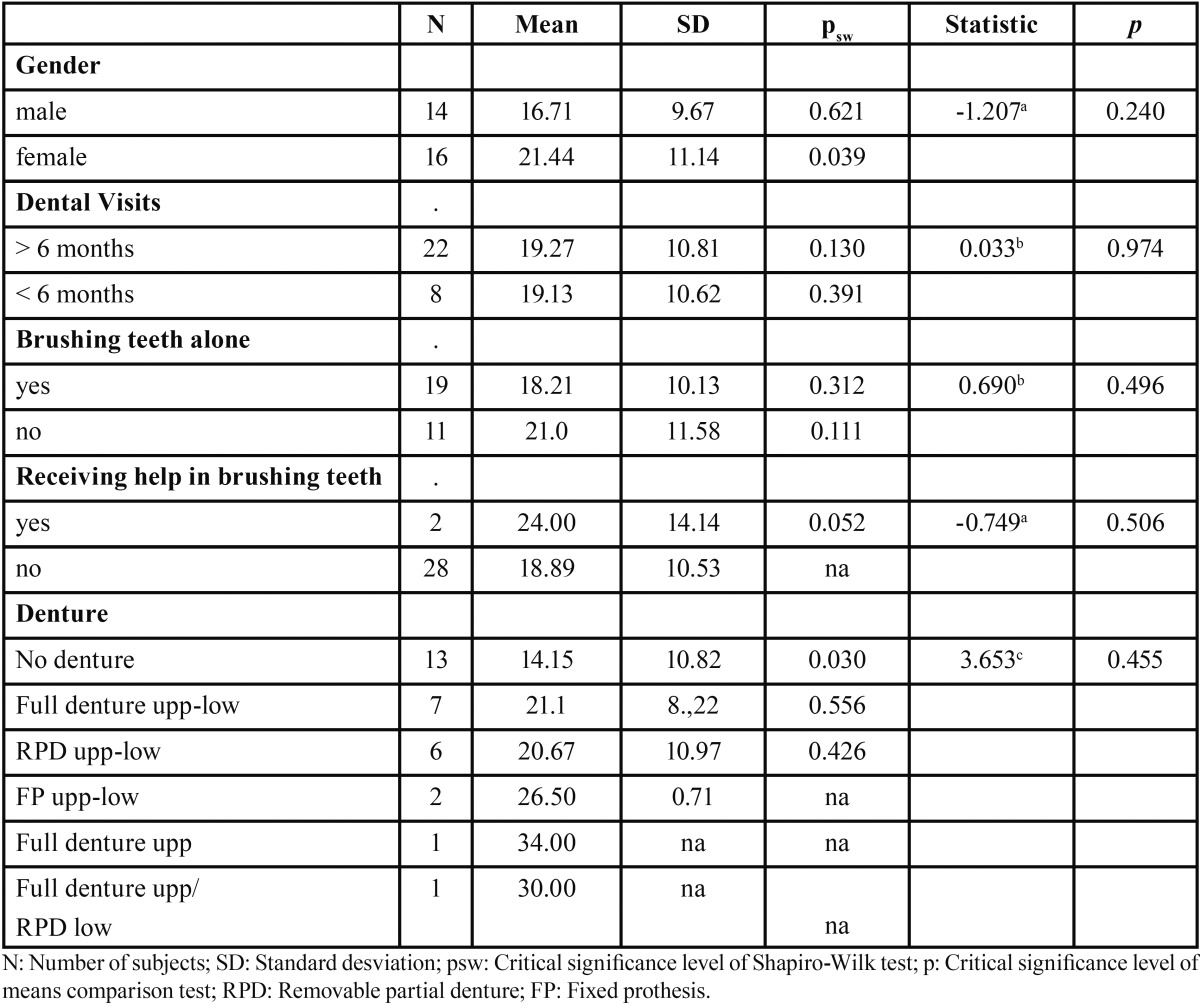
Descriptive statistics, normality tests and t-test for quality of life.

**Table 3:**
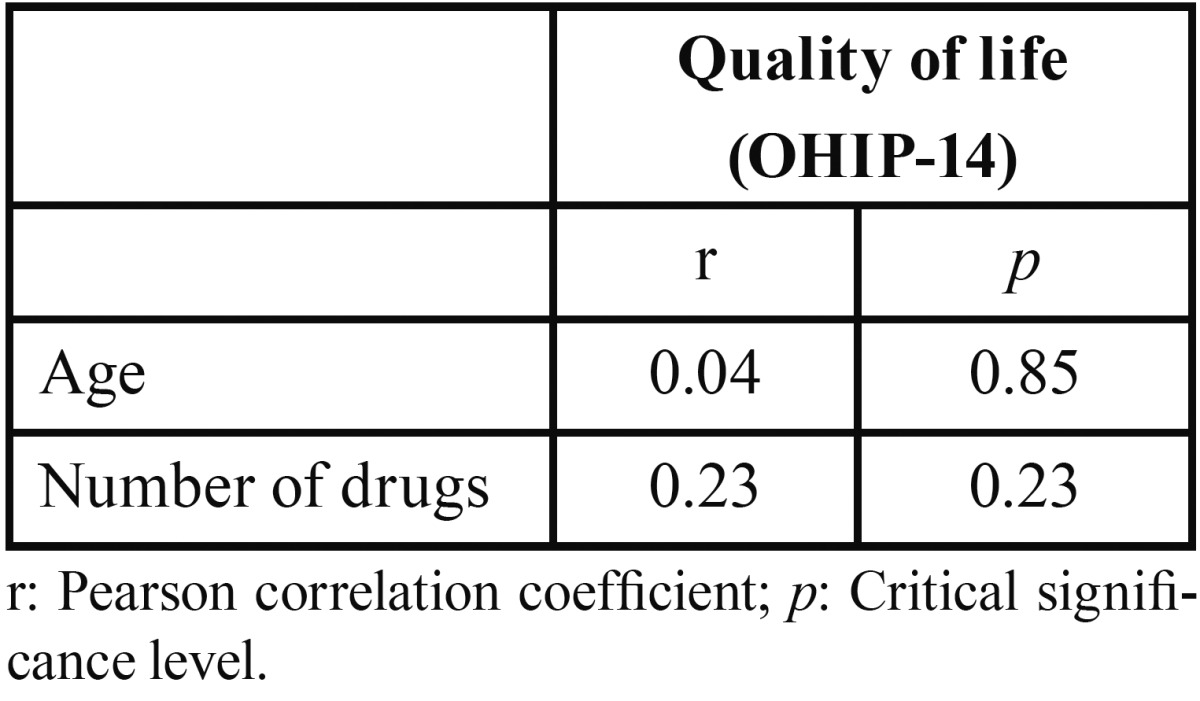
Correlation coefficients (quality of life, age and numbers of drugs).

-Relationship between quality of life and number of remaining teeth and xerostomia: The Pearson correlation test showed that quality of life is not related to the number of remaining teeth. However, it was found that a poorer quality of life is associated with a statistically significant level of xerostomia ( Table 4).

**Table 4:**
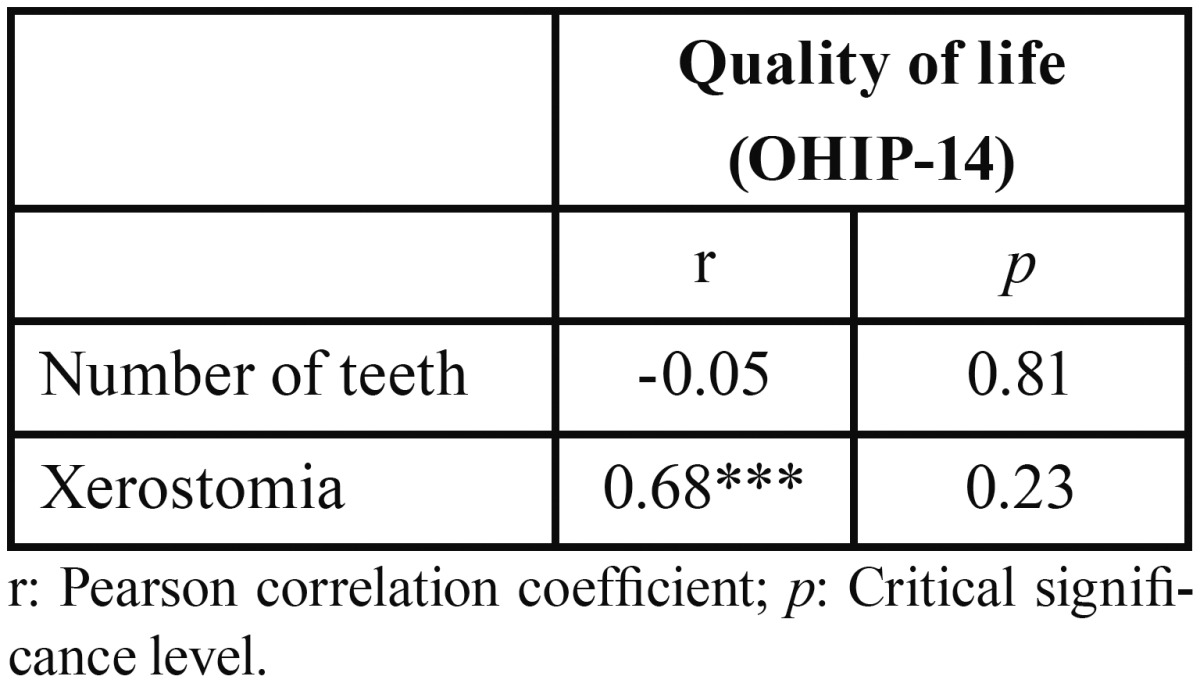
Correlation coefficients (quality of life, number of teeth and xerostomia).

-Relationship between drugs intake and xerostomia: No relationship between the number of drugs taken and level of xerostomia was found.

-Measures to alleviate xerostomia: In order to reduce dry mouth sensation 21 (70%) subjects assured to drink plenty of water, 15 (50%) were taking sugarless candies, 3 (10%) preferred sugar candies, only one (3.3%) person used xerostomia pills from the pharmacy, three (10%) used a specific xerostomia toothpaste, one (3.3%) person used chewing gum and four (13.3%) of them drank unsweetened juice and water to decrease the sensation of dry mouth. Only six (20%) patients did not take anything to alleviate xerostomia.

## Discussion

Life expectancy is increasing as a result of advances in medicine and other social and economic factors, leading to an aging population (15). Aging is accompanied by a gradual physiological deterioration which drives to a reduction in daily activities. The oral functions are also affected, such as the salivary secretion, which results in xerostomia and tooth loss, influencing directly the strength of chewing and swallowing (16). Elderly living in nursing homes are the most vulnerable group to suffer from oral problems. Moreover, these people are often polymedicated, that causes xerostomia. In addition, if oral hygiene is compromised, the risk of oral problems like tooth decay or periodontal disease is increased (17,18).

Quality of life related to oral health is important in dentistry to measure the impact of oral health on patient’s quality of life. In our study the OHIP-14 was chosen to measure quality of life as it is a specific oral test to measure the impact of oral disorders. No significant differences were found in order to assess the quality of life in comparison to age and sex (*p* = 0.853 for age *p* = 0.240 for sex). Therefore, the levels of quality of life are similar in terms of age and sex, consistent with most similar studies.

In our study, the percentage of people receiving help in brushing teeth is low (6.7%), compared with 72.2% of subjects helped by nurses in the Zuluaga *et al.* study, which may result in a higher prevalence of oral lesions (19).

Regarding dental visits, 73.3% (22 people) of subjects did not go for a dental revision for 6 or more months and only did so when they had problems with teeth or dentures. The remaining eight assured going every six months for dental review. In the study performed by Matthews *et al.*, 42% of subjects had made their last dental visit more than five years ago (18). According to our results, the quality of life is not influenced by autonomous toothbrushing (*p* = 0.496) or dental visits (*p* = 0.94), perhaps because of being so spaced in time.

-Number of teeth and prosthesis: The presence of teeth not only helps chewing, but is also involved in pronunciation and helps to have a good facial appearance (20). Akifusa *et al.* postulated that older people with more than 20 teeth are more physically active than those with less than 19 teeth (21). A low number of teeth is associated with a decreased ability to chew food, which decreases the quality of life (22). According to our results, quality of life is not related to the number of remaining teeth. These results disagree with most previous studies. Akifusa *et al.* (21) achieved a better quality of life in subjects with more than 20 teeth. Saintrain and de Souza *et al.* (11) evaluated the impact of tooth loss in 72 edentulous subjects, concluding that it significantly influences the person´s quality of life, especially when it affects their comfort, appearance and nutrition. Christensen *et al.* (17) also confirm the existence of linear association between tooth loss and OHIP. Supporting our results, Kshetrimayum *et al.* argue that subjective oral health and quality of life related to oral health does not always correlate with dental clinical findings (13). Meanwhile, Singh *et al.* (8) verified that having a reduced number of teeth or a low number of posterior teeth does not mean making dietary restrictions that may influence nutrition. The type of prosthesis, according to our study, has no effect on quality of life values (*p* = 0.455). However, Stenman *et al.* found a relationship between the use of prosthesis and quality of life (12).

The increasing number of older people also increase the risk of suffering from diseases, which also increases the number of drugs ingested to combat them. This can affect the salivary glands function, changing the saliva composition as well as salivary flow rate (23). In our study the average drug intake is 8.47 ± 4.55 (being one the minimum and 17 the maximum). This range is similar to that in other studies, as Baker *et al.*, whose range is 1-16 (24).

**Figure 3:**
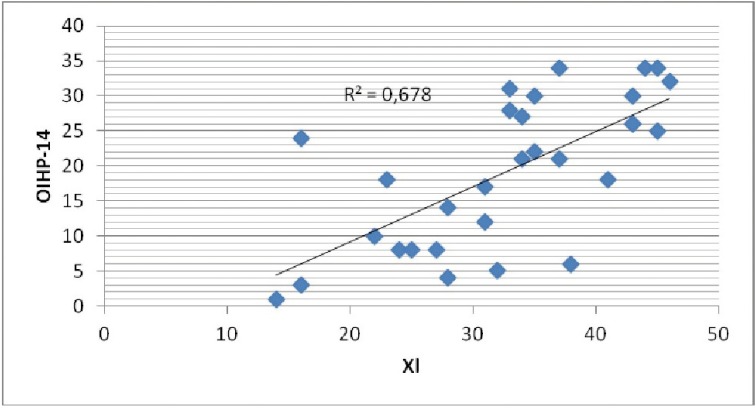
Scatterplot of the association between quality of life (OHIP-14) and xerostomia (XI).

There are many studies linking xerostomia with drugs intake. Närhi *et al.* (25) saw that elderly patients that do not ingest any drugs had a higher salivary flow rate than medicated people. They also showed that the daily intake of more than four drugs reduced salivary flow. In our study, 76.67% of subjects were taking more than four drugs per day. The mean of xerostomia index is 32.77 ± 9.11, indicating an intermediate dry mouth sensation. The correlation between xerostomia and quality of life was positive in our study (Fig. 3). It was verified that the higher XI index score, the poorer quality of life. These results agree with those obtained in the study performed by Baker et al. (24), in which people with poorer quality of life had higher levels of xerostomia. Willumsen *et al.* (26) also found no association between both parameters. However, the number of drugs ingested does not affect the quality of life in our study.

Lack of knowledge on techniques to alleviate dry mouth has been proved as a problem among the elderly. Many of them reported dry mouth sensation, but most had never thought about whether they had mouth with little or too much saliva. 70% of them stated to drink water to compensate it and only a few used symptomatic medication or specific toothpaste for xerostomia. 20% of subjects did not use any measure when dry mouth was noticed (2). Most of our sample (27 out of 30, 76,67%) believe to have a good or acceptable quality of life, with a score lower than 30. Our values are similar to those found in other studies. Baker *et al.* (24) evaluated quality of life in patients with xerostomia. The average life quality was superior to previous studies, being 18.9 for OHIP-14 and 6.2 for the OHIP. Willumsen *et al.* (26) obtained an average quality of life of 5.45 ± 7.25, being slightly lower than our results. In terms of quality of life related to age, no significant differences were found (*p* = 0.853), as in this study.

To conclude, we can say that quality of life is not related to the number of remaining teeth nor the number of ingested drugs. However, a higher level of xerostomia was significantly associated with a poorer quality of life. There is no association between the number of drugs ingested and xerostomia index. Sugarless candies and drinking water are the more frequently used measures to alleviate dry mouth. Due to these results, a good oral health is essential in elderly, so that they can enjoy the best possible quality of life.
